# Anxiety and Depression Disorder Among Young Cannabis Users in Tunisia

**DOI:** 10.1192/j.eurpsy.2022.2147

**Published:** 2022-09-01

**Authors:** S. Brahim, M. Henia, A. Haj Mohamed, M. Chabbouh, L. Zarrouk

**Affiliations:** 1 University Hospital of Mahdia, Psychiatry, chebba, Tunisia; 2 University Hospital of Mahdia, Psychiatry, Mahdia, Tunisia; 3 University Hospital of Mahdia, Tunisia., Psychiatry, mahdia, Tunisia

**Keywords:** Anxiety, Cannabis, Depression

## Abstract

**Introduction:**

The use of cannabis is likely to increase as regulations on its consumption are diminishing throughout the world.

**Objectives:**

to identify the prevalence of anxiety and depression symptoms in a group of cannabis users in Tunisia.

**Methods:**

this a transversal descriptive study about 137 participants in the University Hospital Of Mahdia during 2 months.

**Results:**

In our study population , the consumers were young adults aged between 18 and 35 years old ,of whom 40.8% were professionally active, 23.2% had psychiatric history. Moreover, the use of other substances was regular among users as follows: tobacco among 74.6% of users, alcohol among 72.5% of users, ecstasy among 41.3% of users, cocaine among 25.4% of users. The use of cannabis was considered as a means of exultation for 66.7%, as an anxiolytic for 26.8% and as a sedative for 23.9%. Overall, the effect of cannabis use on anxiety and depression on the HAD scale showed the following results: probable anxiety in 53% of cases, probable state of depression in 72% of time.

**Conclusions:**

The correlation between cannabis use, anxiety and depression remains unclear. Equally concluded, the assumption of self-medication by cannabis stills a topic of discussion.

**Disclosure:**

No significant relationships.

